# Optimal selection of molecular descriptors for antimicrobial peptides classification: an evolutionary feature weighting approach

**DOI:** 10.1186/s12864-018-5030-1

**Published:** 2018-09-24

**Authors:** Jesus A. Beltran, Longendri Aguilera-Mendoza, Carlos A. Brizuela

**Affiliations:** Computer Sciences Department, Center for Scientific Research and Higher Education of Ensenada (CICESE), Carretera Ensenada-Tijuana No. 3918, Zona Playitas, Ensenada, 22860 Mexico

**Keywords:** Antimicrobial peptides, Feature weighting, Molecular descriptors, Classification, Multi-objective evolutionary algorithm, Peptide representation

## Abstract

**Background:**

Antimicrobial peptides are a promising alternative for combating pathogens resistant to conventional antibiotics. Computer-assisted peptide discovery strategies are necessary to automatically assess a significant amount of data by generating models that efficiently classify what an antimicrobial peptide is, before its evaluation in the wet lab. Model’s performance depends on the selection of molecular descriptors for which an efficient and effective approach has recently been proposed. Unfortunately, how to adapt this method to the selection of molecular descriptors for the classification of antimicrobial peptides and the performance it can achieve, have only preliminary been explored.

**Results:**

We propose an adaptation of this successful feature selection approach for the weighting of molecular descriptors and assess its performance. The evaluation is conducted on six high-quality benchmark datasets that have previously been used for the empirical evaluation of state-of-art antimicrobial prediction tools in an unbiased manner. The results indicate that our approach substantially reduces the number of required molecular descriptors, improving, at the same time, the performance of classification with respect to using all molecular descriptors. Our models also outperform state-of-art prediction tools for the classification of antimicrobial and antibacterial peptides.

**Conclusions:**

The proposed methodology is an efficient approach for the development of models to classify antimicrobial peptides. Particularly in the generation of models for discrimination against a specific antimicrobial activity, such as antibacterial. One of our future directions is aimed at using the obtained classifier to search for antimicrobial peptides in various transcriptomes.

**Electronic supplementary material:**

The online version of this article (10.1186/s12864-018-5030-1) contains supplementary material, which is available to authorized users.

## Background

Antimicrobial peptides (AMPs) are components of the host defense mechanism against bacteria and fungi, including multi-drug resistant pathogens such as Methicillin-resistant Staphylococcus aureus and vancomycin-resistant Enterococci [1]. AMPs also exhibit other biological properties like antitumor, antiviral, and antiparasitic activities. With the rapid increase in number of antibiotic-resistant bacteria, AMPs have received much attention as a template for the development of new drugs for the treatment of infectious diseases.

From the computational point of view, Virtual Screening (VS) [2–4] is usually applied at early stages of the drug discovery process. It contributes to the identification of putative AMPs from large peptide libraries [3, 5]. In this context, Quantitative Structure-Activity Relationship (QSAR) is of great importance for models’ generation to classify active (AMPs) and inactive (non-AMPs) peptides [6]. QSAR modeling defines mathematical relationship between the peptides’ physicochemical properties (molecular descriptors) to their biological activity [6] to classify the activity of new peptides. Machine learning approaches are tools for the generation of models that describe this relationship from a set of peptides with known activities. Admittedly, the model’s performance depends on the selection of molecular descriptors since they define the chemical space in which each peptide is projected. The selection of appropriate molecular descriptors to discriminate between AMPs and non-AMPs is a hard goal to achieve due to the large number of molecular descriptors that can be calculated in peptides and to their complex interrelationships. Furthermore, new features can be added to this large set of molecular descriptors through feature construction methods [7]. Recently, an evolutionary approach [8] was proposed for AMP recognition which combines sequence-base features such as motif and positional sequence into more complex features leading to promising results. These results motivate the inclusion of these new features to the existing set to participate in the feature selection process afterwards.

In earlier studies, the selection of molecular descriptors has often been made based on chemical intuition or observed properties that give rise to the antimicrobial activity [3, 9]. In contrast, recent works employ hand-picked features (molecular descriptors) procedures or filtering methods that independently evaluate the features according to a given criterion to select the top *k* of them [8–11]. However, these approaches present some disadvantages considering that the biological activity of peptides depends on complex interrelationships of many molecular descriptors. Therefore, we need a more rigorous feature selection procedure to improve the performance of AMPs classification [12].

Feature selection methods can be categorized into three major classes based on the features’ assessment: filter, wrapper, and hybrid. First, in the filter methods, the quality of features is evaluated from the data, ignoring the effect of the selected features on the classifier algorithm performance [13]. Examples of evaluation functions used on filter methods are distance, information, and dependence measure [14]. Second, the wrapper methods incorporate the classifier’s performance (e.g., error rate, accuracy) to evaluate the quality of the selected features [13]. Finally, hybrid methods combine both, the filter and wrapper methods [15]. Wrappers usually outperform filter methods, mainly because the selection of optimal features is biased towards the effect of these features on the classifier’s performance. Additionally, wrapper methods have a high computational cost because they require to induce and test a classifier for each evaluated features’ subset. In contrast, since filter methods are independent of classification algorithms, they may be computed efficiently [13]. Furthermore, filter methods can improve their performances by using evaluation measures for a specific classification algorithm [13]. For example, the intra-class distance could be appropriate for the instance-based learning algorithms, whereas the information gain for the decision trees classifiers.

An efficient and effective filter approach for the selection of features, based on their weighting has been recently proposed [16]. In this approach, the weights are assigned in such a manner that objects in different classes tend to be far away from each other, whereas objects within the same class tend to be close together. Unfortunately, there is a trade-off between these distances and, that is why the feature weighting challenge is modeled as a multi-objective optimization problem. In a recent work [17] we applied this formulation to the antimicrobial peptides classification problem and improved it by taking into account that molecules with similar structure tend to possess similar biological activity [18]. The central idea is that it makes no sense to minimize the distance among non-AMPs as it should be done [16], since they may have different biological activities. The proof-of-concept of our formulation in [17] showed a good performance capability for the binary classification of AMPs. The present work builds upon our improved formulation [17] and extends its results. Besides dealing with a significantly larger dataset, the statistical significance of the observed difference is assessed. We now also show the ability of our proposal to classify a subset of AMPs that explicitly targeted bacteria.

### Problem statement

The general problem to solve is referred to as feature weighting problem [19], and it is known to be NP-Hard [20]. For our purposes, we model this problem as a multi-objective optimization problem (MOP) to find a set of weight vectors that simultaneously minimize the distance between AMPs and maximize the distances between AMPs and non-AMPs. To define the MOP, we follow a similar approach to the one presented in [16], where the main differences are as follows: first, the general problem of weighting feature in [16] simultaneously minimizes the intra-class distance for all classes. Instead, our approach [17] minimizes only the intra-class distance of AMPs, since the non-AMPs set might contain peptides with different biological activities, thus trying to reduce the intra-class distance for non-AMP would be contradictory with the similarity property principle [18]. Furthermore, in our approach, the number of non-zero weights are used as a tiebreaker criterion for the weight vectors with the same intra or inter-class distances.

#### Notation and definitions

Before presenting the formal definition of the problem, some notation, and definitions are introduced.

- $\mathcal {X}$ is a feature set {*X*_1_,…,*X*_*m*_}. In this paper, we use, without distinction, the term molecular descriptor and feature.
- $\mathcal {Y}$ is the set of class labels {*C*_1_,…,*C*_*c*_}, with *c* the number of classes. For instance, $\mathcal {Y}=\{C_{1}, C_{2}\}$, with *C*_1_= “AMP” and *C*_2_= “Non-AMP”.
- $\mathcal {D}$ is the training dataset composed of *n* peptides with a known biological activity {(**x**_1_,*y*_1_),…,(**x**_*n*_,*y*_*n*_)}, where **x**_*i*_ is an *m*-dimensional vector [*x*_*i*1_,…,*x*_*im*_]^*T*^ that captures the physicochemical properties into real values, each component *x*_*ij*_ encodes the value for the *jth* molecular descriptor (i.e., feature) of the *ith* peptide sequence. $y_{i} \in \mathcal {Y}$ denotes whether **x**_*i*_ has the antimicrobial activity or not. $\mathcal {D}$ can be expressed as a matrix with *n* x (*m*+1) elements whose rows are given by $\mathbf {x}_{i}^{T}$ and *y*_*i*_.
  1 $$ \mathcal{D} = \left[\begin{array}{cccc|c} x_{11} & x_{12} & \cdots & x_{1m} & y_{1}\\ x_{21} & x_{22} & \cdots & x_{2m} &y_{2}\\ \vdots & & \ddots & \vdots & \vdots\\ x_{n1} & x_{n2} & \cdots & x_{nm} & y_{n}\\ \end{array}\right]  $$
  This data matrix $\mathcal {D}$ is also known as a descriptor matrix [21].
- **w**=[*w*_1_,…,*w*_*m*_]^*T*^ is a weight vector that specifies the rescaling value of each feature, the corresponding weight for the *ith* feature is given by [16]:
  2 $$ w_{i} =\left\{ \begin{array}{ll} [\!1,\mathcal{A} ] & \text{if the feature \(X_{i}\) is selected};\\ 0 & \text{if the feature \(X_{i}\) is rejected}. \end{array} \right.  $$
  where $\mathcal {A}$ is the maximum weight for *w*_*i*_ and it takes any positive real number. As in [16], $\mathcal {A}=10$ in this work.
- The weighted distance (also known as weighted Manhattan distance) between two data points **x**_*p*_ and **x**_*q*_ is defined as:
  3 $$  d\left(\mathbf{w}, \mathbf{x}_{p}, \mathbf{x}_{q} \right)=\sum\limits_{i=1}^{m}w_{i}|x_{pi}-x_{qi}|  $$
  where |.| represents the *L*_1_ norm. Let *y*=*AMP* the class label of interest, then the intra-class distance for the class of interest is defined as follows:
  4 $$ D_{intra} \left(\mathbf{w},\mathcal{D} \right) = \sum\limits_{p=1}^{n-1} \sum\limits_{q=p+1 \atop y_{p},y_{q}=AMP}^{n} d(\mathbf{w}, \mathbf{x}_{p}, \mathbf{x}_{q})  $$
  Additionally, the inter-class distance is defined as:
  5 $$ D_{inter}(\mathbf{w},\mathcal{D}) = \sum\limits_{p=1}^{n-1} \sum\limits_{q=p+1\atop y_{p} \neq y_{q}}^{n} d(\mathbf{w}, \mathbf{x}_{p}, \mathbf{x}_{q})  $$

#### A multi-objective approach to the feature weighting problem

Let $\mathcal {D}$ be a training dataset with *n* instances and *m* candidate input features, we assume that for each instance $\mathbf {x}_{i}^{T} \in \mathcal {D}$, the value *x*_*ij*_ is in the interval $[\!1, \mathcal {A}]$, where *x*_*ij*_ is the *j*-th component of the vector $\mathbf {x}_{i}^{T} $. Then, the multi-objective feature weighting problem can be stated as:

6 $$ \begin{aligned} & \underset{\mathbf{w}}{\text{minimize}} & & F(\mathbf{w}) = \left[f_{1}(\mathbf{w}),f_{2}(\mathbf{w})\right]^{T}\\ & \text{subject to} & &w_{i} \in \{0\} \cup [\!1, \mathcal{A}] \; i = 1, \ldots, m,\\ \end{aligned}  $$

where,

$$\begin{array}{*{20}l} f_{1} (\mathbf{w}) &= D_{intra}(\mathbf{w},\mathcal{D})+ \frac{\left[min\{1,\mathbf{w}\}\right]^{T} \mathbf{1}}{m},\\ f_{2}(\mathbf{w}) &= -D_{inter}(\mathbf{w},\mathcal{D}) + \frac{\left[min\{1,\mathbf{w}\}\right]^{T} \mathbf{1}}{m}, \end{array} $$

here, the term [ *min*{1,**w**}]^*T*^**1** is the number of weights that are different from zero (i.e., *w*_*i*_>0 for *i*=1,…,*m*.). This term promotes a weight vector with a smaller number of features than any other weight vector with the same intra-class or inter-class distances.

## Results

To evaluate the effectiveness of our approach, called Multi-Objective Approach for Feature Weighting (MOEA-FW), we conducted experiments on six high-quality benchmark datasets that have recently been used for empirical evaluation of state-of-art antimicrobial prediction tools in an unbiased manner [12]. These datasets were selected because they are composed of manually curated and experimentally validated AMPs; in these datasets, the non-AMPs have the same peptide distribution as that observed in AMPs (see “Methods” section). This experimental study was divided into four parts. In the first part, we aimed at selecting the appropriate molecular descriptors for each dataset through their scaling. Whereas, in the second part, different classification models are induced by four machine learning algorithms (MLAs) with the transformed datasets. In the third part, the best classification models generated were used to predict the antimicrobial activity for new peptides sequences, i.e., peptide sequences that have not been used either for obtaining the weight vectors or for the cross-validation test to choose the best classifiers. Finally, we compared our result with those presented in a recent work [12] that evaluates different AMP predictors.

### Performance measure

To compare the best compromise solutions found by our MOEA-FW algorithm, for each dataset, a performance estimation method was employed to evaluate the efficiency of the model to classify antimicrobial peptides. The performance estimation method employed 10-fold Cross-Validation (10-fold CV) as a re-sampling method and a diverse set of evaluation metrics. In the 10-fold CV, the dataset is partitioned into 10 non-empty disjoint subsets (i.e., fold); each subset has roughly equal size. Nine folds are employed for the machine learning algorithm to induce a classifier, and the classifier is tested on the remaining subset, this procedure is repeated ten times. Additionally, the performance of the classifier was estimated by using the average values from the tests. To test the classification performance, the following metrics were used: accuracy, Matthews correlation coefficient, precision, specificity, sensitivity, balance accuracy, and the area under a ROC Curve (AUC). We consider the instances of the class AMP as positive and the instances of the class non-AMP as negative; then the metrics can be formally defined as follows:

- Accuracy (Acc) [22]:
  7 $$ Acc = \frac{TP + TN }{TP + TN + FP + FN }  $$
- Matthews correlation coefficient (MCC) [22]:
  8 $$ {}MCC = \frac{TP \times TN - FN \times FP}{\sqrt[]{(TP+FN)(TN+FP)(TP+FP)(TN+FN)}}  $$
- Precision (Prec):
  9 $$ Prec = \frac{TP}{TP+FP}  $$
- Sensitivity (Sens):
  10 $$ Sens = \frac{TP}{TP+FN}  $$
- Balance Accuracy (Bal Acc) [12]:
  11 $$ Bal Acc = \frac{1}{2} \left(\frac{TP}{TP+FN} \right) + \frac{1}{2} \left(\frac{TN}{TN+FP} \right)  $$

where TP, TN, FP, and FN are the number of true positive, true negative, false positive, and false negative, respectively. Given that the considered datasets are imbalanced classes (i.e., the AMPs and non-AMPs are not represented equally into the datasets), we used the balance accuracy and the AUC to obtain a better measure of the induced-models’ performance.

### Weighting of molecular descriptors

**Fig. 1:**
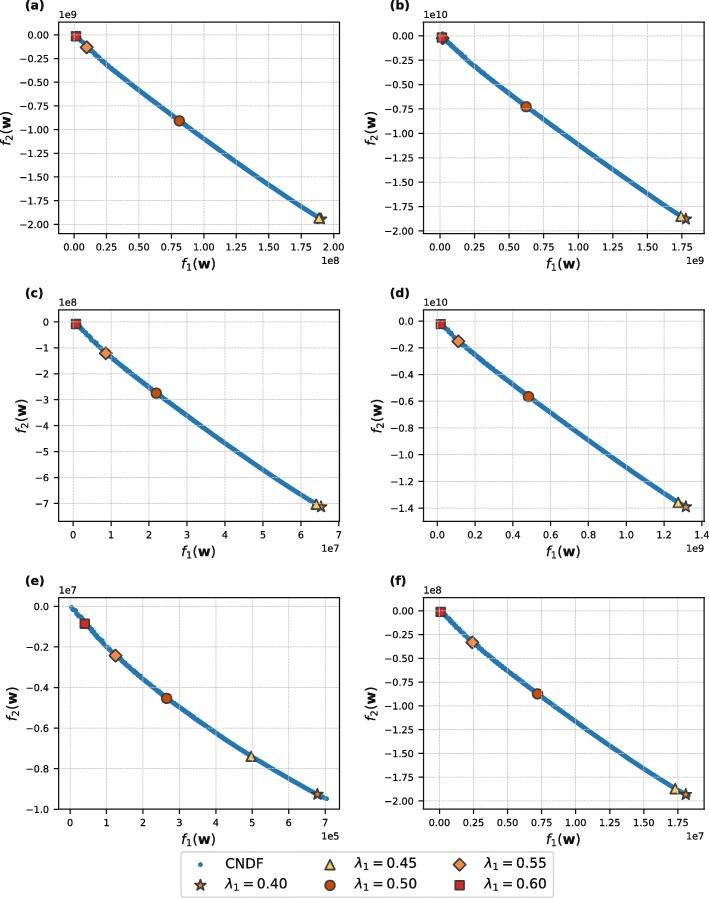
The consolidated non-dominated front (CNDF) visualization. The CNDF is generated after 30 runs of the MOEA-FW approach for each dataset. The markers represent the values for the best compromise solution given *λ*_1_. **a** DAMPD_AMP. **b** APD3_AMP. **c** DAMPD_ANTIBACTERIAL. **d** APD3_ANTIBACTERIAL. **e** DAMPD_BACTERIOCIN. **f** APD3_BACTERIOCIN

Figure 1 displays the consolidated non-dominated front obtained by our approach (MOEA-FW) for each dataset. The consolidated non-dominated front is generated after 30 independent runs of MOEA-FW. The diamond and square marker (i.e., *λ*_1_=0.55 and *λ*_1_=0.6) represent the values for the best compromise solutions that encourage the objective *f*_1_ (i.e., minimize the distance between peptides with antimicrobial activity). Alternatively, *λ*_1_=0.45 and *λ*_1_=0.4 represent the values for the best compromise solutions that encourage the objective *f*_2_ (i.e., maximize the distance between AMPs and non-AMPs). Furthermore, *λ*_1_=0.5 represents the value for the best compromise solution where both objectives are equally important.

**Fig. 2:**
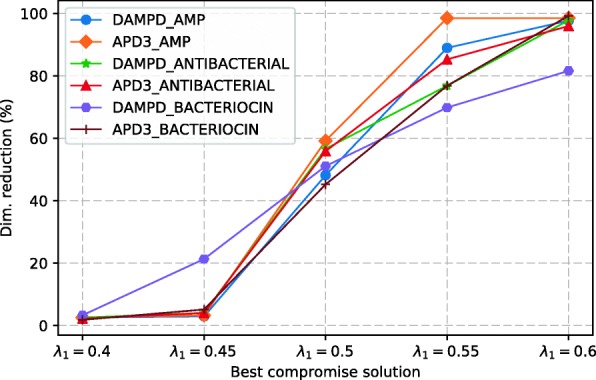
Percentage of number of molecular descriptors reduction for the best compromise solutions on six datasets

The percentage of number of molecular descriptors reduction shown in Fig. 2 indicates a similar behavior on the six datasets for each best compromise solution. In particular, the best compromise solution *λ*_1_=0.5 has, on average, a reduction in the number of molecular descriptors of 52.7%, i.e., on average, the best-weighted solution has 128 features out of 272. Nevertheless, the DAMP_BACTERIOCIN dataset shows an increment of this measure for solution *λ*_1_=0.45. These findings indicate that solutions supporting the objective *f*_1_ (i.e., inter-class distance) have, on average, fewer molecular descriptors than those that support the objective *f*_2_ (i.e., intra-class distance).

### Model selection

Next, for each best compromise solution earlier obtained (i.e., a weight vector **w**), the original datasets were transformed (i.e., weighted; see “Methods” section). Then, for each transformed data, four classification models were constructed by the following machine learning algorithms: random forest (RF), k-nearest neighbor (KNN), multi-layer perceptron (MLP), and a linear support vector machine (SVM-L).

As mentioned earlier, the balance accuracy (*BalAcc*) was considered as a measure to determine the best model on the six datasets weighted by the best compromise solutions. We applied the non-parametric Friedman’s test [23] and Nemenyi post hoc test [24] to verify whether there are significant differences among the classifiers’ performance. The Friedman [23] and Nemenyi tests have been widely used in the literature for statistical comparison of classifiers on multiple datasets (the interested reader is referred to [24] for more information about how to perform both tests).

Our results indicated that the best compromise solution, with *λ*_1_=0.5, allows to induce on average, better classification models regardless the machine learning algorithm, the *BalAcc* was 87.52% (see Additional file 1).

The statistical analysis of the MLAs’ performance identified (by the Friedman test) a significant difference in the *BalAcc* ($\chi ^{2}_{f} (3) = 55.2$, *p*-value = 6.224e-12) of the four MLAs on multiple datasets. Our results show that, on average, SVM-L ranked first (with rank 1.23), KNN second (with rank 2.43), RF third (2.63), and MLP fourth (3.7) (see Additional file 1: Table S1). Furthermore, we found that SVM-L performed significantly better than MLP (Nemenyi: *z*=7.4, *p*-value = 8.40e-13), RF (Nemenyi: *z*=4.2, *p*-value = 0.00016), and KNN (Nemenyi: *z*= 3.6, *p*-value = 0.00181). Similarly, KNN performed significantly better than MLP (*z* = 3.8, *p*-value = 0.00083). Although the KNN performs a little better than RF, there was no statistical significant difference (*p*-value = 0.932) between them.

In particular, considering only the best compromise solution with *λ*_1_=0.5, the average of *BalAcc* for SVM-L was 92.65% and for KNN 90.13% (Summary statistics on *BalAcc*(*%*) for all best compromise solutions can be found in the Additional file 1). Hence, our findings indicate that for the six datasets, the best compromise solution with *λ*_1_=0.5 using SVM-L and KNN induced better classification models.

Table 1 summarizes the result obtained by SVM-L and KNN with the best compromise solution at *λ*_1_=0.5 (detailed results are presented in Additional files 2, 3, 4, 5, 6 and 7). The metric’s values represent the average for the 10-fold cross-validation. In this table, a Wilcoxon test is also performed on the observed differences between KNN and SVM-L for Sens(%), Spec(%), Prec(%), BalAcc, Acc(%), MCC, and AUC values; if the difference is statistically significant, at a confidence level of 95%, then an asterisk is added to the winner value (in bold). In most cases, the classification models generated by the KNN showed better specificity and precision than the ones generated by the SVM-L, i.e., models correctly predict 96% of non-AMPs, and correctly classify 80% of predicted AMPs. In comparison, the classification model obtained by SVM-L showed good sensitivity, namely, the model correctly classifies 88.33% of AMPs.

**Table 1:** 10-Fold Cross-Validation performance on six datasets for KNN and SVM-L, *λ*_1_=0.5

Dataset	MLA	Sens(%)	Spec(%)	Prec(%)	Bal Acc(%)	Acc(%)	MCC	AUC
DAMPD_AMP	KNN	71.97	**9** **7** **.** **2** **2** ^∗^	**8** **3** **.** **7** **5** ^∗^	84.60	**9** **3** **.** **0** **1**	**0** **.** **7** **3** **5**	0.846
	SVM-L	**8** **8** **.** **0** **7** ^∗a^	92.30	69.56	**9** **0** **.** **1** **9** ^∗^	91.62	0.734	**0.90**2^∗^
APD3_AMP	KNN	80.85	**95.2**7^∗^	**7** **7** **.** **2** **3** ^∗^	88.06	**9** **2** **.** **8** **5**	0.747	0.881
	SVM-L	**9** **1** **.** **6** **5** ^∗^	92.53	70.75	**9** **2** **.** **0** **9** ^∗^	92.36	**0** **.** **7** **6** **2**	**0** **.** **9** **2** **1** ^∗^
DAMPD_ANTIBACTERIAL	KNN	**9** **1** **.** **0** **4**	96.45	**8** **4** **.** **3** **7**	**9** **3** **.** **7** **5**	**9** **5** **.** **5** **1**	**0** **.** **8** **4** **9**	**0** **.** **9** **3** **7**
	SVM-L	88.49	**9** **6** **.** **5** **4**	84.18	92.51	95.06	0.832	0.925
APD3_ANTIBACTERIAL	KNN	79.32	**9** **5** **.** **3** **0** ^∗^	**7** **7** **.** **1** **8** ^∗^	87.31	**9** **2** **.** **6** **1**	0.738	0.873
	SVM-L	**9** **1** **.** **3** **4** ^∗^	92.22	70.33	**9** **1** **.** **7** **8** ^∗^	92.07	**0** **.** **7** **5** **6**	**0** **.** **9** **1** **8** ^∗^
DAMPD_BACTEROCIN	KNN	100	95.53	85.83	97.76	96.36	0.902	0.978
	SVM-L	100	**9** **8** **.** **8** **9**	**9** **6** **.** **6** **7**	**9** **9** **.** **4** **4**	**9** **9** **.** **0** **9**	**0** **.** **9** **7** **7**	**0** **.** **9** **9** **4**
APD3_BACTEROCIN	KNN	83.50	**9** **5** **.** **0** **4**	77.05	89.27	93.12	0.758	0.893
	SVM-L	**8** **5** **.** **3** **8**	94.83	**7** **7** **.** **2** **8**	**9** **0** **.** **1** **0**	93.12	**0** **.** **7** **6** **8**	**0** **.** **9** **0** **1**

Each value is the average performance from 10-fold cross-validation by the classifier built by the machine learning algorithm (second column) on the dataset (first column). Wilcoxon signed rank test was performed on the measure resulting from the 10-fold cross-validation of KNN and SVM-L. The models with significant improvement at *p*-value ≤0.05 are marked with the symbol *

^a^Bold font indicates the best value per measure for every dataset

**Fig. 3:**
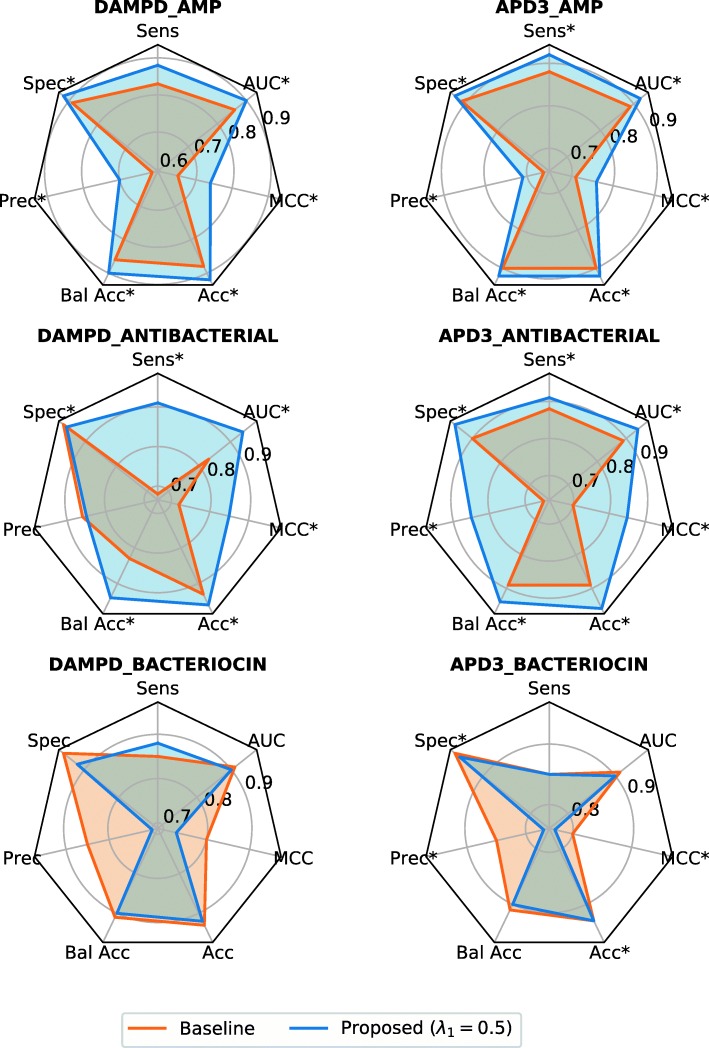
Performance comparison between the best model achieved by MOEA-FW and the baseline. Each plot shows the performance measure by 10-fol cross-validation of the best model achieved by MOEA-FW and the baseline (i.e., all candidate input features) for a particular dataset. The polygon represents a particular performance’s model. When a polygon is covered means that the model is worse in all metrics that the model represented by the polygon that includes it. Wilcoxon signed rank test was performed on the measure resulting from the 10-fold cross-validation of best model achieved by MOEA-FW and the baseline. The models with significant improvement at *p*-value ≤0.05 are marked with the symbol *

To determine the effect of MOEA-FW on the efficiency of the model to classify AMPs for each dataset, we compared the performance of two classifiers generated by the same machine learning algorithm, one applying the MOEA-FW and the other one, by using all candidate input features (i.e., baseline). We selected the best machine learning algorithm per database, this is according to the balanced accuracy column in Table 1. We run the Wilcoxon’s test on the *BalAcc* resulting from the 10-fold cross-validation of our proposed method and the baseline for each dataset (Additional files 2, 3, 4, 5, 6 and 7). The models generated by MOEA-FW shows a significant improvement over the baseline models on the *BalAcc*. For each dataset, the significant difference in *BalAcc* between MOEA-FW and baseline were as follows: DAMPD_AMP (*p*-value = 0.00976), APD3_AMP (*p*-value = 0.00195), DAMPD_ANTIBACTERIAL (*p*-value = 0.00195), APD3_ANTIBACTERIAL (*p*-value = 0.00976), DAMPD_BACTERIOCIN (*p*-value = 0.051), and APD3_ BACTERIOCIN (*p*-value = 0.08398). Similar results were observed for the other metrics, they are summarized in Fig. 3. In this figure, an asterisk indicates that the observed different is statistically significant.

On the other hand, if we take into consideration other metrics (i.e., Sens, Spec, AUC, MCC, Pres, Acc) to compare both models, the results show that the models generated by using MOEA-FW achieve a comparable or superior performance than those obtained by using all candidate input features. In particular, for the datasets DAMPD_AMP, APD3_AMP, and APD3_ANTIBACTERIAL, the MOEA-FW shows an improvement over the baseline (see Fig. 3). In contrast, datasets DAMPD_BACTERIOCIN and APD3_BACTERIOCIN showed a decrease in the precision measure with respect to the baseline. This result suggests that our proposal cannot find a suitable chemical space for BACTERIOCIN datasets, whereby an efficient model could be induced to discriminate what a bacteriocin is. Conjectures of why this is happening are given in the “Discussion” section.

### Model assessment

After selecting the best models obtained with the best compromise solution given *λ*_1_=0.5, and using KNN and SVM-L, we measured their prediction capacity over new peptide sequences, this is, peptide sequences that have not been used either for obtaining the weight vectors or for cross-validation tests to choose the best classifiers (see “Methods” section). We observed that all classifiers induced by SVM-L have an AUC value >0.83, this means that the models generated by SVM-L have an excellent capacity to learn what an antimicrobial peptide is. Whereas, the model generated by KNN maintain an excellent specificity (as the results presented in Table 1 indicate).

On the other hand, comparing the results for DAMP_BACTIBASE set, espacially for bacteriocin, in Tables 1 and 2, the considerable difference in sensitivity (Sens(%)) may be because of the small number of bacteriocins in the test set.

**Table 2:** Performance comparison of KNN and SVM-L on unseen sequences from the six datasets, *λ*_1_=0.5

Dataset	ML	Sens(%)	Spec(%)	Prec(%)	Bal Acc(%)	Acc(%)	MCC	AUC
DAMPD_AMP	KNN	72.16	**9** **4** **.** **1** **7**	**6** **8** **.** **6** **3**	83.17	**9** **0** **.** **8** **7**	**0** **.** **6** **5** **0**	0.832
	SVM-L	**7** **7** **.** **3** **2** ^a^	91.62	61.98	**8** **4** **.** **4** **7**	89.47	0.631	**0** **.** **8** **4** **5**
APD3_AMP	KNN	70.82	**9** **2** **.** **1** **1**	**6** **5** **.** **1** **0**	81.47	**8** **8** **.** **4** **5**	**0** **.** **6** **0** **9**	0.815
	SVM-L	**8** **9** **.** **2** **4**	82.87	51.98	**8** **6** **.** **0** **5**	83.97	0.597	**0** **.** **8** **6** **1**
DAMPD_ANTIBACTERIAL	KNN	**8** **0** **.** **0**	90.91	60.27	**8** **5** **.** **4** **5**	89.30	0.634	**0** **.** **8** **5** **5**
	SVML	74.55	**9** **3** **.** **1** **0**	**6** **5** **.** **0** **8**	83.82	**9** **0** **.** **3** **7**	**0** **.** **6** **4** **0**	0.838
APD3_ANTIBACTERIAL	KNN	65.97	**9** **3** **.** **9** **1**	**6** **8** **.** **3** **5**	79.94	89.26	0.607	0.799
	SVM-L	**8** **1** **.** **9** **4**	91.55	65.92	**8** **6** **.** **7** **5**	**8** **9** **.** **9** **5**	**0** **.** **6** **7** **6**	**0** **.** **8** **6** **7**
DAMPD_BACTEROCIN	KNN	**8** **0**	87.50	50.00	**8** **3** **.** **7** **5**	86.49	0.561	**0** **.** **8** **3** **8**
	SVM-L	60	**9** **6** **.** **8** **8**	**7** **5** **.** **0** **0**	78.44	**9** **1** **.** **8** **9**	**0** **.** **6** **2** **6**	0.784
APD3_BACTEROCIN	KNN	75.86	**9** **4** **.** **2** **3**	70.97	85.05	91.35	0.682	0.850
	SVM-L	**9** **3** **.** **1** **0**	92.95	**7** **1** **.** **0** **5**	**9** **3** **.** **0** **3**	**9** **2** **.** **9** **7**	**0** **.** **7** **7** **4**	**0** **.** **9** **3** **0**

^*^Each value is the performance on the testing dataset by the classifier built by the machine learning algorithm (second column) on the dataset after applying the best compromise solution for *λ*_1_=0.5 (first column)

^a^Bold font indicates the best value per measure for every dataset

### Comparison with existing AMP classifiers

The best model generated by our approach MOEA-FW was compared with others AMP predictors that used the same datasets. It is important to note that the number of instances between our test and the test showed in [12] are different, because in [12] the evaluation of AMP’s predictors was performed by using the full examples of the six datasets, whereas in our method, we used only 20% of them (i.e., the other 80% of the dataset was used in the optimization process, see “Methods” section). However, this comparison is intended to observe the predictive capacity of the classification models generated with our approach and those presented by the state-of-the-art methods.

The classifier performances presented in this work and those reported by state-of-art methods for the AMP prediction are summarized in Tables 3 and 4. Our results reflect that the models produced by our approach have a better performance than the state-of-the-art methods for the classification of antimicrobial and antibacterial peptides. It is worth noting that models derived from our approach to classify antibacterial peptides outperformed AntiBP [25] and AntiBP2 [26] (see Tables 3 and 4). However, our method is improved by BAGEL3 [27] for the BACTERIOCIN datasets.

**Table 3:** Performance comparison among the AMPs prediction methods reported in [12] with our proposed approach for the DAMPD dataset

Tool	Task	Sens(%)	Spec(%)	Prec(%)	Bal Acc(%)
MOEA-FW(SVM-L)	Antimicrobial	77.32	91.62	**6** **1** **.** **9** **8**	**8** **4** **.** **4** **7**
CAMPR3(RF)		**9** **2** **.** **3** **2** ^a^	72.65	40.30	82.49
CAMPR3(SVM)		90.13	72.10	39.25	81.11
ADAM		84.09	68.88	35.09	76.49
MLAMP		63.62	82.27	41.78	72.94
DBAASP		22.12	**9** **2** **.** **8** **7**	38.28	57.49
AMPA		48.81	84.79	39.09	66.80
MOEA-FW(KNN)	Antibacterial	80.00	**9** **0** **.** **9** **1**	**6** **0** **.** **2** **7**	**8** **5** **.** **4** **5**
AntiBP		**8** **9** **.** **7** **8**	45.05	24.63	67.41
AntiBP2		86.90	15.97	17.14	51.44
MOEA-FW(KNN)	Bacteriocin	80.00	87.50	50.00	83.75
BAGEL3		**9** **3** **.** **5** **5**	**1** **0** **0** **.** **0**	**1** **0** **0** **.** **0**	**9** **6** **.** **7** **7**
BACTIBASE		83.87	**1** **0** **0** **.** **0**	**1** **0** **0** **.** **0**	91.93

^a^Bold font indicates the best value per measure

**Table 4:** Performance comparison among the AMPs prediction methods reported in [12] with our proposed approach for the APD3 dataset

Tool	Task	Sens(%)	Spec(%)	Prec(%)	Bal Acc(%)
MOEA-FW(SVM-L)	Antimicrobial	89.24	82.87	**5** **1** **.** **9** **8**	**8** **6** **.** **0** **5**
CAMPR3(RF)		**9** **4** **.** **8** **0** ^a^	72.65	40.30	82.49
CAMPR3(SVM)		90.60	72.10	39.25	81.11
ADAM		91.07	68.88	35.09	76.49
MLAMP		75.59	82.27	41.78	72.94
DBAASP		62.81	92.87	38.28	57.49
AMPA		39.17	**8** **4** **.** **7** **9**	39.09	66.80
MOEA-FW(SVM-L)	Antibacterial	**8** **1** **.** **9** **4**	**9** **1** **.** **5** **5**	**6** **5** **.** **9** **2**	**8** **6** **.** **7** **5**
AntiBP2		66.59	26.00	15.25	46.30
MOEA-FW(SVM-L)	Bacteriocin	**9** **3** **.** **1** **0**	92.95	71.05	93.03
BAGEL3		86.36	**1** **0** **0** **.** **0**	**1** **0** **0** **.** **0**	**9** **3** **.** **1** **8**
BACTIBASE		38.36	**1** **0** **0** **.** **0**	**1** **0** **0** **.** **0**	69.48

^a^Bold font indicates the best value per measure

## Discussion

Our approach aims to identify a weight for each molecular descriptor, in such manner that, peptides with antimicrobial activity tend to be close together, whereas peptides with different biological activities tend to be far away from each other. Our results indicate that the best compromise solution with *λ*_1_=0.5 allows, on average, the best balance accuracy for all six databases. Furthermore, this solution allows a reduction of at least 52% in the number of molecular descriptors. It is important to note that in our previous work [17], the best solution, for a smaller database, was found with *λ*_1_=0.55, and it reduced the number of descriptors by 67.90%. The difference may be a consequence of having unbalanced datasets in this case. With the best compromise solution (*λ*_1_=0.5), we transform (weight the features) the datasets and build models for the binary classification of AMPs and non-AMPs. Our results indicate that both KNN and SVM-L allow to achieve reliable models for classification of antimicrobial and antibacterial peptides. These results support the idea that our MOEA-FW approach allows generating better models for a specific antimicrobial activity, in this particular case, antibacterial activity. In this direction, we expect to use this approach in the future, to classify other specific antimicrobial activities, such as antiviral, anti-fungal, and anti-parasitic, accordingly to determine whether this classification performance is also observed in those particular antimicrobial activities.

As mentioned earlier, the models generated by KNN achieve high specificity and precision, while models induced by SVM-L produce high sensitivity (see Table 1). These results suggest that, combining the models generated by KNN and SVM-L, we could exploit their properties to generate even more efficient models.

On the other hand, the lowest performance model generated by MOEA-FW was for the classification of peptides which source and target are bacteria (i.e., bacteriocins). In this case, our approach was not able to produce a chemical space where both, the peptide activity and their source could be discriminated. It is important to note, that BAGEL3 [27] and BACTIBASE [28] use properties related to sequence similarity to classify bacteriocins.

## Conclusions

This work deals with the problem of molecular descriptors weighting by modeling it as a multi-objective optimization problem, such that peptides with different biological activities tend to be far away from each other, whereas, AMPs tend to be close together. To solve this problem, a variant of a general methodology [16] based on a multi-objective evolutionary algorithm (MOEA/D-DE) [29, 30] was employed. Also, we introduce a multi-criteria decision-making approach to select the weight vectors with different degrees of satisfaction between the intra-class and inter-class distances for the target class. Then, with these weight vectors, we scaled the datasets where the peptides are represented by molecular descriptors, and generated different models for the binary classification of AMPs. The analysis of experimental results, on six unbalanced datasets, indicates that the proposed methodology is effective on the development of models to predict antimicrobial peptides. Particularly, in the generation of models for discrimination against a specific antimicrobial activity, such as antibacterial. Given this last result, future research direction aims at constructing classifiers that specialize in specific antimicrobial activities such as antiviral, antifungal, antitumor, among others.

## Methods

**Fig. 4:**
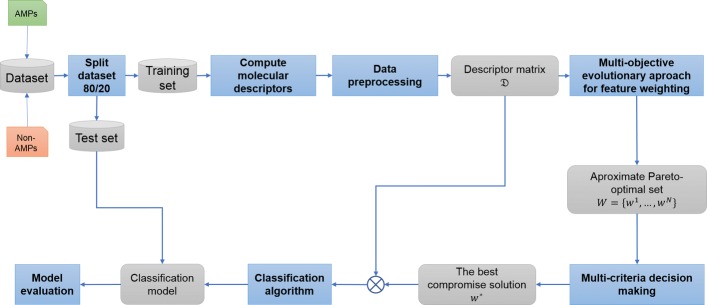
The overall scheme of the feature weighting framework. The rectangles with bold texts represents processes, and the rounded rectangles represent the inputs and outputs of processes

The scheme of the methodology adopted in this study is shown in Fig. 4. Each process is described in detail in this section, including selection and splitting of a dataset, computing and preprocessing of molecular descriptors, molecular descriptor weighting, and classification of antimicrobial peptides.

### Data collection

For this study, we used six sets of peptide sequences, for which AMPs are experimentally validated whereas non-AMPs were randomly selected from a supersequence generated from the concatenation of proteins retrieved from UniProt. None of the retrieved proteins have been annotated as antimicrobial, and some of them are intracellular. From the supersequence, six non-AMPs are randomly extracted for each AMP in the dataset [12]. The datasets were obtained from the publicly available supplementary data of a recent work [12]. Then, we removed the peptide sequence that contains non-standard residues (e.g., peptide sequences with undetermined amino acids such as ’X’, ’B’, ’J’ or ’Z’). We named these datasets according to i) the database from which the AMPs were recovered and, ii) their annotated activity. Regarding their database, we named the datasets DAMPD and APD3, because they come from the Dragon Antimicrobial Peptide Database (DAMPD) [31] and the Antimicrobial Peptide Database (APD3) [32], respectively. Regarding their annotated activity, we named the datasets as AMP, ANTIBACTERIAL and BACTERIOCIN. AMP are peptides that have antimicrobial activity. ANTIBACTERIAL is a proper subset of AMP since they are antimicrobial peptides that explicitly targeted bacteria. Additionally, BACTERIOCIN is a proper subset of ANTIBACTERIAL, the source organisms of such peptides are also bacteria (these peptides are referred to as bacteriocins, the interested reader is referred to [33] for more information on peptide naming and classification).

Each dataset was split into two random parts, training and test sets. The training set contains 80% of randomly selected sequences from the original dataset, while the test set contains the remaining sequences (see Table 5). The training set is used in the next steps of this section, while the test set is only used to test the effectiveness of the models generated by our approach.

**Table 5:** Summary of peptide datasets

Dataset	No. of AMP sequences	No. of Non-AMP sequences	Total
DAMPD_AMP	438	2174	2612
DAMPD_ANTIBACTERIAL	255	1242	1497
DAMP_BACTEROCIN	24	123	147
APD3_AMP	1360	6860	8220
ADP3_ANTIBACTERIAL	1158	5777	6935
ADP3_BACTEROCIN	125	612	737

^*^The datasets were extracted from [12] and we removed the sequences with non-standard residues

### Computation of molecular descriptors

Molecular descriptors are derived from a logical and mathematical procedure which transform physical and chemical information encoded in a molecule representation into useful numbers [34]. Nowadays, there are many proposed descriptors, that can be grouped according to their dimensionality from 0D to 3D. The 0D descriptors are very simple molecular properties (e.g., molecular mass and atom count), that depend only on the molecular composition of the peptide. The 1D descriptors encode information about molecular structural fragments (e.g., distance between two cysteine residues, hydrophobic moment). The 2D descriptors are also known as topological descriptors, and they give us information contained in a molecular graph (e.g., Weiner index). Furthermore, 3D descriptors capture the molecular geometry, stereochemical, and surface properties [6].

Two free software packages were used to extract molecular descriptors: Tango [35–37] and the in-house Java Peptide Descriptor from Sequences (JPEDES) tool [17]. The first one was used to compute the following physicochemical properties: *α*-helix propensity, *β*-sheet propensity, turn structure propensity, and in vitro aggregation. Whereas, JPEDES [17] was used to codify OD and 1D descriptors. Unfortunately, the 3D descriptors were not computed due to unavailability 3D-structures for most known AMPs. Altogether four molecular descriptors were computed using Tango [35–37] and other 268 with JPEDES tool [17]. Those descriptors were extracted for each peptide sequence in the training and test datasets.

### Preprocessing

We conducted a two-level preprocessing for the descriptor matrix previously generated. First, we applied a preprocessing at the instance level that consisted of removing outliers; these are vectors labeled with the same class that are very different from the rest, and that might affect the performance of chemical space characterization. Second, we applied a preprocessing at the descriptor level that renders all molecular descriptor values to the same range. This is because the employed molecular descriptors have different range values, e.g., the isoelectric point takes values in the order of 10^0^ to 10^1^ pH units, whereas the molecular weight in the order of 10^2^ to 10^3^ Daltons.

To remove isolated vectors concerning their neighborhood, the Local Outlier Factor (LOF) [38] method was used. It should be noted that the LOF was applied to each class (e.g., AMP and non-AMP) from each dataset. Regarding preprocessing at the descriptor level, we applied the Min-Max scaling method, which maps the measures for each descriptor into a range of 1 to 10 [17]. As a result, we obtained a normalized descriptor matrix $\mathcal {D}$.

### Multi-objective evolutionary approach for feature weighting (MOEA-FW)

The multi-objective evolutionary algorithm based on decomposition (MOEA/DDE) [29, 30] was employed to solve the multi-objective feature weighting problem earlier formulated (see Problem statement). The work in [30] shows that MOEA/D-DE performs better than the well known NSGAII [39] for continuous optimization problems, like the one described in this study (see Eq. 6).

In short, MOEA/D-DE decomposes the multi-objective optimization problem into *N* single-objective optimization problems by adopting the Tchebycheff approach. Then, these N problems are solved simultaneously (for a detailed description of this method we refer to the interested reader to [29, 30]).

In general, this algorithm receives as input the descriptor matrix $\mathcal {D}$ and gives as output a set of approximated *N* optimal solutions to (6), this is called approximate Pareto set: $\mathcal {P^{*}}=\left \{\mathbf {w^{1}}, \ldots, \mathbf {w^{N}}\right \}$. It should be noted that each solution is a weight vector $\mathbf {w^{k}} =\left [w_{1}^{k}, \ldots, w_{m}^{k}\right ]^{T}$, where the *i*-th component is the scale factor for the *i*-th molecular descriptor. For each solution *w*^*k*^ in $\mathcal {P^{*}}$, an objective vector *F*(*w*^*k*^)=[*f*_1_(*w*^*k*^),*f*_2_(*w*^*k*^)]^*T*^ is assigned. Then the set of all these objective vectors is called the approximate Pareto front [40]: *PF*={*F*(*w*^1^),…,*F*(*w*^*N*^)}.

**Fig. 5:**
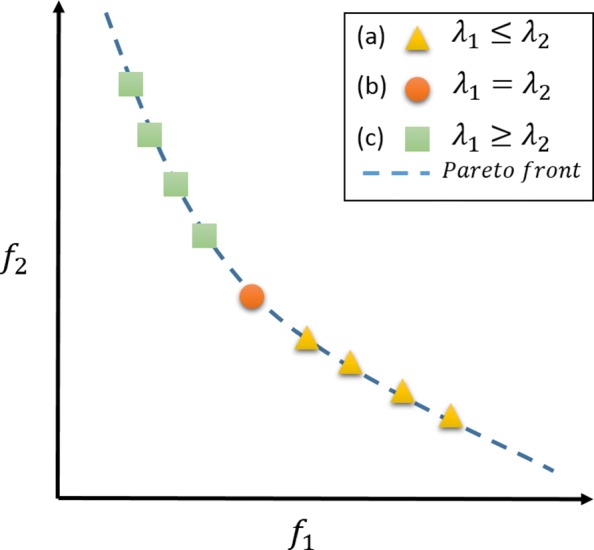
The weighted sum approach. Illustration of the weighted sum approach. (a) *f*_1_ is less important than *f*_2_. (b) *f*_1_ is equally important as *f*_2_. (c) *f*_2_ is less important than *f*_1_

It is important to note that, solutions in $\mathcal {P^{*}}$ cannot be considered better among themselves in both objectives since they are in a trade-off relation. This means that, some solutions in $\mathcal {P^{*}}$ are better in objective *f*_1_ than in *f*_2_ and vice-versa (see Fig. 5). To draw a few solutions from $\mathcal {P^{*}}$, taking into account different satisfiability levels of the objectives, we employed a well-established process in multi-criteria decision making [40].

#### Multi-criteria decision making approach to select weight vectors

For the problem of choosing a few weight vectors from the approximate Pareto set $\mathcal {P^{*}}$, we followed a process that receive as input $\mathcal {P^{*}}$. The main steps can be described as follows:

- **Step 1:** for each solution, $\mathbf {w} \in \mathcal {P^{*}}$, scale the values for objective functions *f*_1_(**w**) and *f*_2_(**w**) to a range between 0 and 1, where 1 means full satisfaction for a particular objective, and 0 indicates dissatisfaction. Perform the scaling of a solution *w*^*k*^ for the objective *f*_*i*_ as follows [16]:
  12 $$ \mu_{i}^{k} =\left\{ \begin{array}{ll} 1 & \text{if } f_{i}\left(\mathbf{w^{k}}\right) = f_{i}^{\min},\\ \frac{f_{i}^{\max} - f_{i}\left(\mathbf{w^{k}}\right)}{f_{i}^{\max} -f_{i}^{\min}} & \text{if } f_{i}^{\min} < f_{i}^{k} < f_{i}^{\max},\\ 0 & \text{if } f_{i}\left(\mathbf{w^{k}}\right) = f_{i}^{\max}, \end{array} \right.  $$
  where,
  13 $$\begin{array}{*{20}l} f_{i}^{\min} &= \underset{ 1 \leq j \leq N }{\min} \left\{f_{i}\left(\mathbf{w^{j}}\right) \right\}, \end{array} $$
  14 $$\begin{array}{*{20}l} f_{i}^{\max} &= \underset{ 1 \leq j \leq N }{\max} \left\{f_{i}\left(\mathbf{w^{j}}\right) \right\}. \end{array} $$
  Here $\mathbf {\mu ^{k}} = \left [\mu _{1}^{k},\mu _{2}^{k}\right ]^{T}$ is the objective vector constrained to the [0,1] range for the solution *w*^*k*^ in the approximate Pareto set $\mathcal {P^{*}}$.
- **Step 2:** perform a weighted sum approach given a weight vector **λ**= [ *λ*_1_,*λ*_2_]^*T*^. Here *λ*_1_ and *λ*_2_ are used to set the preference over objectives *f*_1_ and *f*_2_, respectively. For instance, if we want a solution that satisfies *f*_1_ more than *f*_2_, then a greater value should be assigned to *λ*_1_ than to *λ*_2_ (see Fig. 5). Given **λ**, the weighted sum for each objective vector *μ*^*k*^ is calculated as follows:
  15 $$ g^{bcs}(\mathbf{\mu}| \lambda_{1}) = \lambda_{1} \mu_{1} + (1-\lambda_{1}) \mu_{2}  $$
- **Step 3:** Find the best compromise solution given **λ**, namely, the weight vector **w**^*k*∗^ with the maximum value of *g*^*bcs*^ (formally described in Eq. 1).
  16 $$ k^{*}=\underset{k \in [1,N]}{\arg \max} \;\;\; g^{bcs}\left(\mathbf{\mu^{k}}| \lambda_{1}\right)  $$

In this work, for each dataset, we selected five of the best compromise solutions by using *λ*_1_ equals to 0.4, 0.45, 0.5, 0.55, and 0.60.

### Classification algorithms

This section describes an assessment method to validate the performance of the MOEA-FW method. In this method, we evaluated the classification task before (i.e., baseline), and after applying our MOEA-FW algorithm. For each classification task, we built four models: random forest (RF), k-nearest neighbor (KNN), a linear support vector machine (SVM-L), and a multi-layer perceptron (MLP). A training dataset without weight factors was used before applying our MOEA-FW algorithm, and the weighted molecular descriptors are used after that. Later, we compared the classification performance between the models and measured indirectly the quality of our proposal. To accomplish this, each best compromise solution (i.e., the weight vector *w*^*k*∗^) was applied to dataset $\mathcal {D}$ resulting in a new dataset $\mathcal {\hat {D}}_{\mathbf {W}}$, where:

17 $$ \mathcal{\hat{D}}_{\mathbf{W}} = \left[\begin{array}{cccc|c} w_{1}x_{11} & w_{2}x_{12} & \cdots & w_{m}x_{1m} & y_{1}\\ w_{1}x_{21} & w_{2}x_{22} & \cdots & w_{m}x_{2m} & y_{2}\\ \vdots & & \ddots & \vdots & \vdots\\ w_{1}x_{n1} & w_{2}x_{n2} & \cdots & w_{m}x_{nm} & y_{n}\\ \end{array}\right]  $$

In this way, after applying our proposal, the rejected molecular descriptors correspond to columns whose values are zero and those columns were deleted.

### Implementation details

All experiments were performed under the following condition; OS: ubuntu 16.04 LTS; CPU: Intel i7 at 2.40GHz; and RAM memory: 12 GB.

The MOEA/D-DE algorithm was implemented in Java using the framework of Metaheuristics for solving multi-objective optimization problems MOEA Framework 2.1 (available from http://www.moeaframework.org). The main parameters for MOEA/D-DE were set according to the values recommended in [30] for 2-objective problems, the specific parameter settings are summarized in [17].

The classification algorithms were implemented in Python 3.6 using the Scikit-learn [41]. Scikit-learn is an efficient set of tools for the implementation of machine learning algorithms for data mining tasks. The machine learning algorithms’ hyperparameters are summarized as a following: KNN (*p*=1,*weight*=*distance*) and *k*=19,22,3 for the antimicrobial, antibacterial, and bacteriocin datasets, respectively; SVM-L (class_weight= balanced) and the penalty parameter C = 0.001, 0.1, and 0.001 for the antimicrobial, antibacterial, and bacteriocin datasets, respectively; RF (criterion =gini, max_features =sqrt); finally for MLP we used the default hyperparameters.

## Additional files

Additional file 1 Performance comparison of the best compromise solutions given *λ*_1_ for four different machine learning algorithms. The values are related to the average of the balance accuracy. (PDF 341 kb)

Additional file 2 Predictions of antimicrobial activity for DAMPD_AMP. Evaluation of different models with DAMP_AMP after applying the best compromise solutions and four machine learning algorithms. This file shows the results obtained by each of the best compromise solutions given *λ*_1_ in each fold of the 10-fold cross-validation test. (CSV 33 kb)

Additional file 3 Predictions of antimicrobial activity for APD3_AMP. Evaluation of different models with APD3_AMP after applying the best compromise solutions and four machine learning algorithms. This file shows the results obtained by each of the best compromise solutions given *λ*_1_ in each fold of the 10-fold cross-validation test. (CSV 34 kb)

Additional file 4 Predictions of antibacterial activity for DAMPD_ANTIBACTERIAL. Evaluation of different models with DAMPD_ANTIBACTERIAL after applying the best compromise solutions and four machine learning algorithms. This file shows the results obtained by each of the best compromise solutions given *λ*_1_ in each fold of the 10-fold cross-validation test. (CSV 32 kb)

Additional file 5 Predictions of antibacterial activity for APD3_ANTIBACTERIAL. Evaluation of different models with APD3_ANTIBACTERIAL after applying the best compromise solutions and four machine learning algorithms. This file shows the results obtained by each of the best compromise solutions given *λ*_1_ in each fold of the 10-fold cross-validation test. (CSV 34 kb)

Additional file 6 Predictions of bacterocins for DAMPD_BACTERIOCIN. Evaluation of different models with DAMPD_BACTERIOCIN after applying the best compromise solutions and four machine learning algorithms. This file shows the results obtained by each of the best compromise solutions given *λ*_1_ in each fold of the 10-fold cross-validation test. (CSV 22 kb)

Additional file 7 Predictions of bacterocins for APD3_BACTERIOCIN. Evaluation of different models with APD3_BACTERIOCIN after applying the best compromise solutions and four machine learning algorithms. This file shows the results obtained by each of the best compromise solutions given *λ*_1_ in each fold of the 10-fold cross-validation test. (CSV 31 kb)

## Abbreviations

10-fold CV: 10-fold Cross-Validation
Acc: Accuracy
AMPs: Antimicrobial peptides
APD3: Antimicrobial Peptide Database
AUC: Area under a ROC curve
Bal Acc: Balance accuracy
DAMPD: Dragon Antimicrobial Peptide Database
JPEDES: Java Peptide Descriptor from Sequences
KNN: k-nearest neighbor
LOF: Local Outlier Factor
MCC: Matthews correlation coefficient
MLAs: machine learning algorithms
MLP: Multi-layer perceptron
MOEA-FW: Multi-Objective Approach for Feature Weighting
MOEA/D-DE: Multi-objective evolutionary algorithm based on decomposition
MOP: Multi-objective optimization problem
Prec: Precision
QSAR: Quantitative Structure-Activity Relationship
RF: Random forest
Sens: Sensitivity
SVM-L: Linear support vector machine
VS: Virtual Screening
