# Supplementary material for: Optimal selection of molecular descriptors for antimicrobial peptides classification: an evolutionary feature weighting approach
Source: BMC Genomics. 2018 Sep 24;19(Suppl 7):672. doi: 10.1186/s12864-018-5030-1 (PMC6156846; doi:10.1186/s12864-018-5030-1)
Supplement: Supplementary file 1 — Performance comparison of the best compromise solutions given λ1 for four different machine learning algorithms. The values are related to the average of the balance accuracy. (PDF 341 kb) [file 12864_2018_5030_MOESM1_ESM.pdf]

Table S1. Performance comparison of the best compromise solutions given λ1 for four different machine learning algorithms. The ranks in the parentheses are used in computation of the Friedman test.

**Dataset**	**Best Compromise solution**	**Average Bal Acc(%)**				
		**RF (Rank)**	**KNN (Rank)**	**MLP (Rank)**	**SVM-L (Rank)**	**Average**
DAMPD _AMP	λ_1= 0.40	87.42 (2)	83.98 (4)	84.04 (3)	88.94 (1)	86.10
APD3_AMP		89.75 (2)	88.77 (2)	78.77 (3)	92.51 (1)	87.45
DAMPD _ANTIBACTERIAL		88.54 (3)	92.30 (2)	81.37 (3)	94.37 (1)	89.15
APD3_ANTIBACTERIAL		89.00 (2)	88.02 (3)	85.20 (3)	92.21 (1)	88.61
DAMPD_BACTERIOCIN		95.31 (2)	97.64 (1)	60.42 (3)	94.44 (1)	86.95
APD3_BACTERIOCIN		79.74 (4)	87.72 (2)	86.89 (2)	90.43 (1)	86.20
Avg. Bal Acc(%) λ_1=0.40		88.29	89.74	79.45	92.15	87.41
DAMPD _AMP	λ_1 = 0.45	87.52 (2)	84.31 (4)	84.44 (3)	89.15 (1)	86.36
APD3_AMP		88.19 (2)	88.14 (3)	83.68 (4)	92.49 (1)	88.13
DAMPD _ANTIBACTERIAL		89.54 (3)	92.80 (2)	78.70 (4)	93.76 (1)	88.70
APD3_ANTIBACTERIAL		88.11 (2)	87.55 (3)	84.52 (4)	91.85 (1)	88.00
DAMPD_BACTERIOCIN		90.63 (3)	98.26 (1)	68.61 (4)	92.78 (2)	87.57
APD3_BACTERIOCIN		78.39 (4)	87.94 (2)	83.52 (3)	89.16 (1)	84.75
Avg. Bal Acc(%) λ_1=0.45		87.06	89.84	80.58	91.53	87.25
DAMPD _AMP	λ_1= 0.50	86.08 (2)	85.01 (3)	84.13 (4)	89.88 (1)	86.27
APD3_AMP		87.07 (3)	87.68 (2)	82.35 (4)	92.20 (1)	87.32
DAMPD _ANTIBACTERIAL		89.25 (3)	93.75 (1)	77.52 (4)	92.51 (2)	88.26
APD3_ANTIBACTERIAL		87.04 (4)	87.31 (3)	87.74 (2)	91.78 (1)	88.47
DAMPD_BACTERIOCIN		90.63 (3)	97.76 (2)	71.32 (4)	99.44 (1)	89.79
APD3_BACTERIOCIN		77.68 (4)	89.27 (2)	83.02 (3)	90.10 (1)	85.02
Avg. Bal Acc(%) λ_1=0.50		86.29	90.13	81.01	**92.65**	**87.52**
DAMPD _AMP	λ_1 = 0.55	82.26 (3)	83.84 (2)	79.65 (4)	86.83 (1)	83.14
APD3_AMP		82.81 (1)	68.38 (4)	79.13 (2)	75.53 (3)	76.46
DAMPD _ANTIBACTERIAL		89.60 (3)	92.49 (2)	81.33 (4)	93.70 (1)	89.28
APD3_ANTIBACTERIAL		85.65 (3)	86.30 (2)	83.33 (4)	87.83 (1)	85.78
DAMPD_BACTERIOCIN		82.19 (3)	98.26 (2)	69.44 (4)	100.0 (1)	87.47
APD3_BACTERIOCIN		74.16 (3)	86.64 (2)	72.13 (4)	88.71 (1)	80.41
Avg. Bal Acc(%) λ_1=0.55		82.78	85.99	77.50	88.76	83.76
DAMPD _AMP	λ_1= 0.6	72.58 (1)	69.04 (3)	62.93 (4)	72.04 (2)	69.15
APD3_AMP		83.03 (2)	78.69 (3)	58.91 (4)	84.01 (1)	76.16
DAMPD _ANTIBACTERIAL		75.51 (2)	66.29 (3)	60.29 (4)	80.02 (1)	70.53
APD3_ANTIBACTERIAL		78.53 (2)	73.51 (3)	70.46 (4)	83.00 (1)	76.37
DAMPD_BACTERIOCIN		93.75 (3)	97.76 (2)	85.54 (4)	98.33 (1)	93.85
APD3_BACTERIOCIN		68.02 (3)	69.29 (2)	66.24 (4)	78.71 (1)	70.56
Avg. Bal Acc(%) λ_1=0.6		78.57	75.77	67.39	82.68	76.10
Avg. Bal Acc(%) by ML		84.60	86.29	77.19	**89.56**	
**Average rank**		2.63	2.43	3.70	**1.23**	

*The bold values are the highest for each best compromise solution and the machine learning algorithm

Table S2. The critical difference for the average rank between SVM-L (R_svm) and the three remaining MLAs

i	classifier	z=(R_svm - R_i)/SE	p
1	RF	-4.20	0.00016
2	KNN	-3.60	0.00181
3	MLP	-7.40	8.40E-13

Table S3. The critical difference for the average rank between KNN (R_KNN) and the remaining MLAs

i	classifier	z=(R_svm - R_i)/SE	p
1	RF	-0.60	0.93208
2	MLP	-3.80	0.00083

Table S4. The critical difference for the average rank between RF (R_RF) and the remaining MLAs

i	classifier	z=(R_svm - R_i)/SE	p
1	MLP	-3.20	0.00751

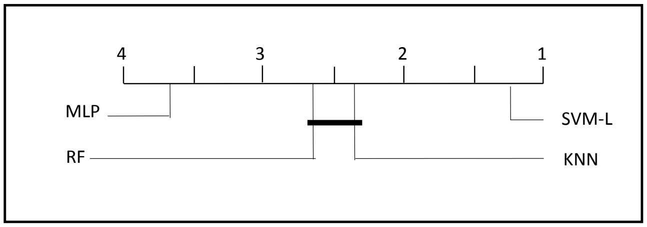

Figure S1. Comparison of the four MLAs on the datasets with the Nemenyi test. Groups of classifiers that are not significantly different (at p=0.10) are connected.
